# Neurobiological Effects of Non-Invasive Behavioural Management Strategies in Paediatric Dentistry: A Scoping Review

**DOI:** 10.3390/clinpract16090160

**Published:** 2026-08-27

**Authors:** Love Bukola Ayamolowo, Idayat Adetoun Raji, Bukola Abimbola Adesoji, Dorcas Oluwatola Adelakun, Moréniké Oluwátóyìn Foláyan

**Affiliations:** 1 Faculty of Nursing Sciences, Obafemi Awolowo University, Ile-Ife 22005, Nigeria; 2 Department of Nursing Services, Obafemi Awolowo University Teaching Hospitals Complex, Ile-Ife 22007, Nigeria; oreadesoji2006@yahoo.co.uk (B.A.A.);; 3 Department of Child Dental Health, Obafemi Awolowo University, Ile-Ife 22005, Nigeria

**Keywords:** behaviour modification, behaviour guidance, psychological interventions, children’s dentistry, non-pharmacological interventions, neurological impact, observational learning, pet therapy

## Abstract

Behavioural management strategies used in paediatric dentistry have neurobiological effects. This scoping review aimed to map the existing literature on the neurobiological effects of non-invasive behavioural management strategies used in paediatric dentistry. The study protocol was registered on the Open Science Framework (registration number: OSF.IO/9EZVT). A search was conducted on PubMed, Web of Science, Scopus, Google Scholar, and African Journals Online (AJOL) for studies published up to May 2025. The review followed the Preferred Reporting Items for Systematic Reviews and Meta-Analyses extension for Scoping Reviews (PRISMA-ScR) guidelines. Studies were included if they assessed non-invasive behavioural strategies in children aged 0–17 years undergoing dental procedures and reported at least one neurobiological or physiological outcome. The data were summarised descriptively, and key findings were thematically synthesised to identify trends in behavioural strategy types and neurobiological outcomes measured. Out of 1720 records retrieved, 35 studies met the inclusion criteria. These included randomised and non-randomised controlled trials and observational studies conducted in 13 countries on five continents involving 2919 children. The behaviour management strategies identified were 18 in number, with distraction (*n* = 19) and tell–show–do (*n* = 17) being the most frequently applied. The neurobiological outcomes assessed included anxiety (*n* = 36), pain perception (*n* = 8), fear (*n* = 7), and stress response (*n* = 1). Their physiological regulation was assessed using heart rate (*n* = 26), oxygen saturation (*n* = 10), blood pressure (*n* = 8), pulse rate (*n* = 6), cortisol (*n* = 4), respiratory rate (*n* = 1), and oxytocin (*n* = 1). Behavioural assessment tools used were classified into Child-Reported Anxiety and Fear Instruments (*n* = 23), Pain Perception Instruments (*n* = 9), Clinician/Observer-Rated Behavioural and Anxiety Scales (*n* = 18), and Parental and Proxy Reports (*n* = 2). This review underscores the emerging evidence that non-invasive behavioural strategies may be linked to neurobiology in paediatric dentistry. More studies are needed in new countries to be able to map the possible impact of context and culture on study findings.

## 1. Introduction

Dental fear is a common occurrence, an essential and inevitable emotion that arises in response to stress induced by various dental procedures [1]. The intensity of dental fear varies from nervousness and anxiety to dental phobia, and it is a barrier to successful completion of treatment [1], thereby compromising oral health [2]. Effective behavioural management of children in dental settings is, therefore, important for ensuring successful treatment outcomes and fostering positive dental experiences from an early age.

The dental clinic can be a source of anxiety and fear for children, due to unfamiliar surroundings, perceived threat of pain, or previous negative experiences. These emotional responses may result in disruptive behaviours that impede treatment and negatively influence long-term oral health attitudes [3]. Consequently, behavioural management techniques have become central to paediatric dental care, enabling clinicians to gain cooperation and reduce distress during procedures [4].

Behavioural management strategies in paediatric dentistry have evolved from traditional authority-based approaches to contemporary patient-centred models [4]. Traditional methods such as tell–show–do, voice control, and protective stabilisation were primarily designed to achieve immediate behavioural compliance and clinical efficiency [5,6]. In contrast, current approaches are grounded in developmental psychology and neuroscience to reduce anxiety and enhance comfort and cooperation by prioritising rapport building, perceived control, and emotional support [7]. This is achieved by using tools like audiovisual distraction, mobile applications, and memory restructuring [7]. These non-invasive techniques are often preferred by caregivers across a range of cultural contexts due to their safety profile, simplicity, and the extent to which they align with child-centred care principles [8,9].

While considerable research has focused on the clinical effectiveness and parental acceptability of behavioural management techniques, there is a growing interest in understanding their neurobiological impact. Behavioural changes are underpinned by physiological mechanisms, including the activation or regulation of stress-response systems in the body and brain [10,11,12]. For instance, during dental procedures, children may exhibit changes in cortisol levels, heart rate variability, and autonomic nervous system (ANS) activity, reflecting activation of the hypothalamic–pituitary–adrenal (HPA) axis and associated neuroendocrine pathways [13,14].

The amygdala serves as the primary neural hub for processing threat-related stimuli and coordinating anxiety responses [15,16]. When activated, it receives threat-related information from cortical, hippocampal, and thalamic pathways, initiating autonomic nervous system responses, including increased heart rate, heightened alertness, and other physiological changes characteristic of anxiety [15]. Techniques that foster a sense of control and predictability—such as tell–show–do and parental presence—are hypothesised to exert their anxiolytic effects, in part, through top-down regulatory mechanisms involving the prefrontal cortex. Specifically, these interventions may enhance prefrontal inhibitory control over amygdala reactivity, thereby attenuating threat perception and dampening downstream stress responses, including reduced cortisol secretion [17,18]. This is consistent with evidence that prefrontal–amygdala connectivity plays a critical role in emotion regulation and that interventions promoting cognitive reappraisal and perceived control can modulate this circuitry [18]. Similarly, activities that foster trust and social bonding, such as animal-assisted therapy, may increase oxytocin release, which has been shown to dampen fear responses and promote cooperative behaviour [7,13].

Despite the theoretical and empirical relevance of these findings, the neurobiological effects of behavioural interventions in paediatric dentistry remain poorly synthesised. Most studies in this area are limited in scope, context, or outcome measures. There is currently no comprehensive review that brings together this emerging body of evidence or evaluates the methodological tools used to study neurobiological responses in dental settings.

This scoping review addresses this gap. It aims to identify and map the available literature on the neurobiological effects of non-invasive behavioural management techniques used in paediatric dentistry using established methodological frameworks for scoping reviews [19,20]. Specifically, it maps publications on the physiological and brain-based responses associated with commonly used behavioural strategies.

## 2. Materials and Methods

This scoping review followed the Preferred Reporting Items for Systematic Reviews and Meta-Analyses extension for Scoping Reviews (PRISMA-ScR) guidelines to ensure a transparent and systematic approach [20]. A review protocol detailing the objectives, inclusion criteria, and methodology was registered on the Open Science Framework (OSF; https://doi.org/10.17605/OSF.IO/9EZVT) (Table S1).

### 2.1. Research Questions

This review was guided by a primary question: What are the neurobiological effects of non-invasive behavioural management strategies used in paediatric dentistry? Secondary questions were as follows: (1) What behavioural strategies were evaluated for neurobiological outcomes? (2) What neurobiological or physiological effects were reported?

A Population, Concept and Context (PCC) framework was used to define the scope of the review [21]. This framework was specifically selected because it aligns with the scoping review methodology and facilitates systematic mapping of population, concept, and context variables that are essential for identifying research gaps in this emerging field. (1) Population: Paediatric dental patients (0–17 years); (2) Concept: Non-invasive behavioural management strategies (e.g., tell–show–do, distraction, virtual reality (VR), modelling, aromatherapy, animal-assisted therapy) and their neurobiological or physiological effects (e.g., cortisol, heart rate, blood pressure, electroencephalogram (EEG), galvanic skin response (GSR), ANS responses, oxytocin, pain perception biomarkers); and (3) Context: dental procedures within paediatric dentistry settings.

### 2.2. Search Strategy

A comprehensive literature search was conducted across PubMed (MEDLINE), Scopus, Web of Science, Google Scholar, and AJOL up to 28 May 2025. No restrictions were placed on the year of publication, and all studies meeting the predefined eligibility criteria were considered for inclusion. The search strategy combined Medical Subject Headings (MeSH), Emtree terms, and free-text keywords related to paediatric dentistry, behavioural management techniques, and neurobiological or physiological stress responses. Sample search terms included: (“paediatric dentistry” OR “pediatric dentistry”) AND (“behavioural management” OR “non-pharmacological intervention”) AND (cortisol OR EEG OR “heart rate variability” OR “autonomic nervous system”). The search strategy was reviewed using the Peer Review of Electronic Search Strategies (PRESS) checklist [22]. Reference lists of included articles and relevant reviews were hand-searched for additional studies (Table S2).

### 2.3. Title, Abstract, and Article Screening

All retrieved citations were imported into EndNote (version X9) for deduplication and then uploaded to Rayyan for screening. Two reviewers (LBA and DTA) independently screened the titles and abstracts for relevance. Full-text articles were then assessed for eligibility. Records that did not meet the inclusion criteria were removed. The reference list of the records included for screening was examined for any other related publications. Disagreements were resolved by discussion or adjudicated by a third reviewer (MOF). The inter-rater reliability between reviewers was assessed using Cohen’s kappa coefficient.

### 2.4. Eligibility Criteria

Articles were included if they were published in English, peer-reviewed, full-text original research, focused on children aged 0 to 17 undergoing dental procedures, involved non-invasive behavioural management techniques, and reported at least one neurobiological or physiological outcome. Exclusion criteria were the use of pharmacological sedation or invasive behavioural techniques; studies conducted outside of dental healthcare settings; and non-primary research such as reviews, editorials, opinion pieces, commentaries, and conference abstracts.

### 2.5. Data Extraction

A pre-tested standardised data extraction form was used by two reviewers to independently extract the following data: study characteristics (author, year, country, journal), sample size and demographics (age, sex), dental context or procedures involved, behavioural management strategy applied, neurobiological or physiological outcomes reported, measurement tools and methods. Disagreements in the extracted data were resolved through discussion.

### 2.6. Data Analysis

Data were summarised descriptively using frequencies and percentages where appropriate. Key findings were synthesised thematically to identify trends in behavioural strategy types, neurobiological outcomes measured, and instruments used for evaluation. Tables were used to aid the clarity of reporting. As this was a scoping review, no critical appraisal or risk-of-bias assessment of individual studies was conducted.

## 3. Results

**Figure 1:**
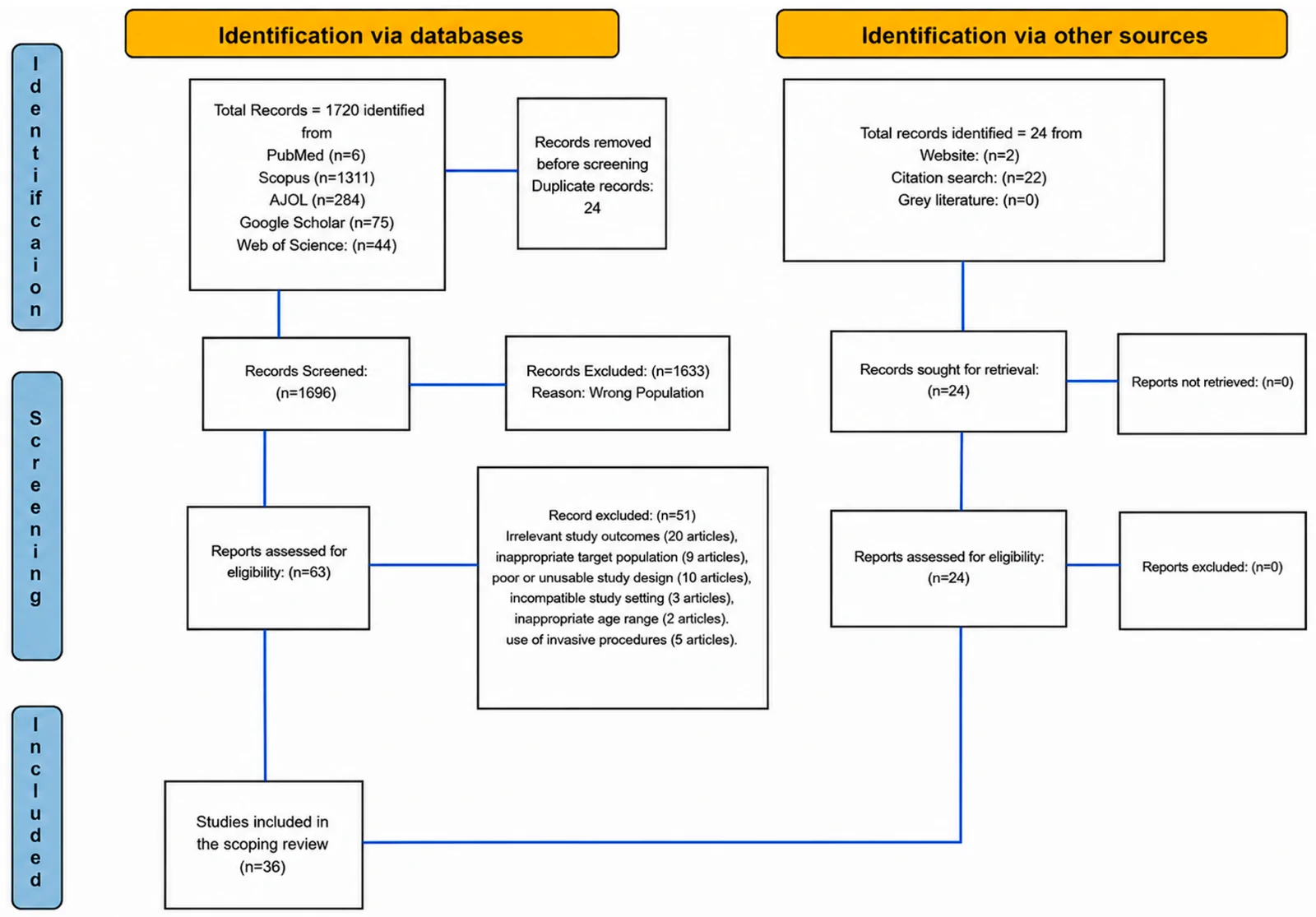
PRISMA flow diagram for the study.

The search yielded 1720 articles from PubMed (*n* = 6), Scopus (*n* = 1311), AJOL (*n* = 284), Google Scholar (*n* = 75) and Web of Science (*n* = 44). The records were reduced to 1696 after removing 24 duplicates. During this initial screening phase, 1633 articles were excluded for not meeting the inclusion criteria, leaving 63 articles for full-text assessment. The 63 full-text articles were thoroughly reviewed, and 51 studies were further excluded for reasons ranging from irrelevant study outcomes (20 articles), to inappropriate target population (9 articles), poor or unsuitable study design (10 articles), incompatible study setting (5 articles), inappropriate age range (2 articles), and use of invasive procedures (5 articles). After applying all exclusion criteria, a total of 12 articles were deemed eligible. An additional 22 articles were also identified via other sources—website (2 articles), citation search (22 articles)—and included. A total of 36 studies were included in the final scoping review [6,23,24,25,26,27,28,29,30,31,32,33,34,35,36,37,38,39,40,41,42,43,44,45,46,47,48,49,50,51,52,53,54,55,56,57]. The selection process is visually summarised in the accompanying PRISMA flow diagram (Figure 1).

### 3.1. Characteristics of Included Studies

Table 1 shows that the 36 studies were conducted across 13 countries on five continents: Asia, Africa, Europe, South America, and North America. The largest concentration of studies was from Asia, with India contributing 18 studies [23,24,25,26,27,28,29,30,31,32,33,34,35,36,37,38,39], Saudi Arabia contributing two [40,41], two from Thailand [42,57], and one each from Iran [43], Pakistan [44], and Syria [45]. From Africa, Egypt had three studies [46,47,48]. In Europe, two studies were from Turkey [49,50], one from Italy [51], one from Spain [52], and another from Switzerland [53]. In South America, two studies were from Brazil [54,55]. North America was represented by two studies from the United States [6,56]. The number of children recruited for the studies was 2919, with sample sizes ranging from a single-case report [56] to a study involving 400 participants [38]. Participants’ ages ranged from 3 to 17 years.

**Figure 2:**
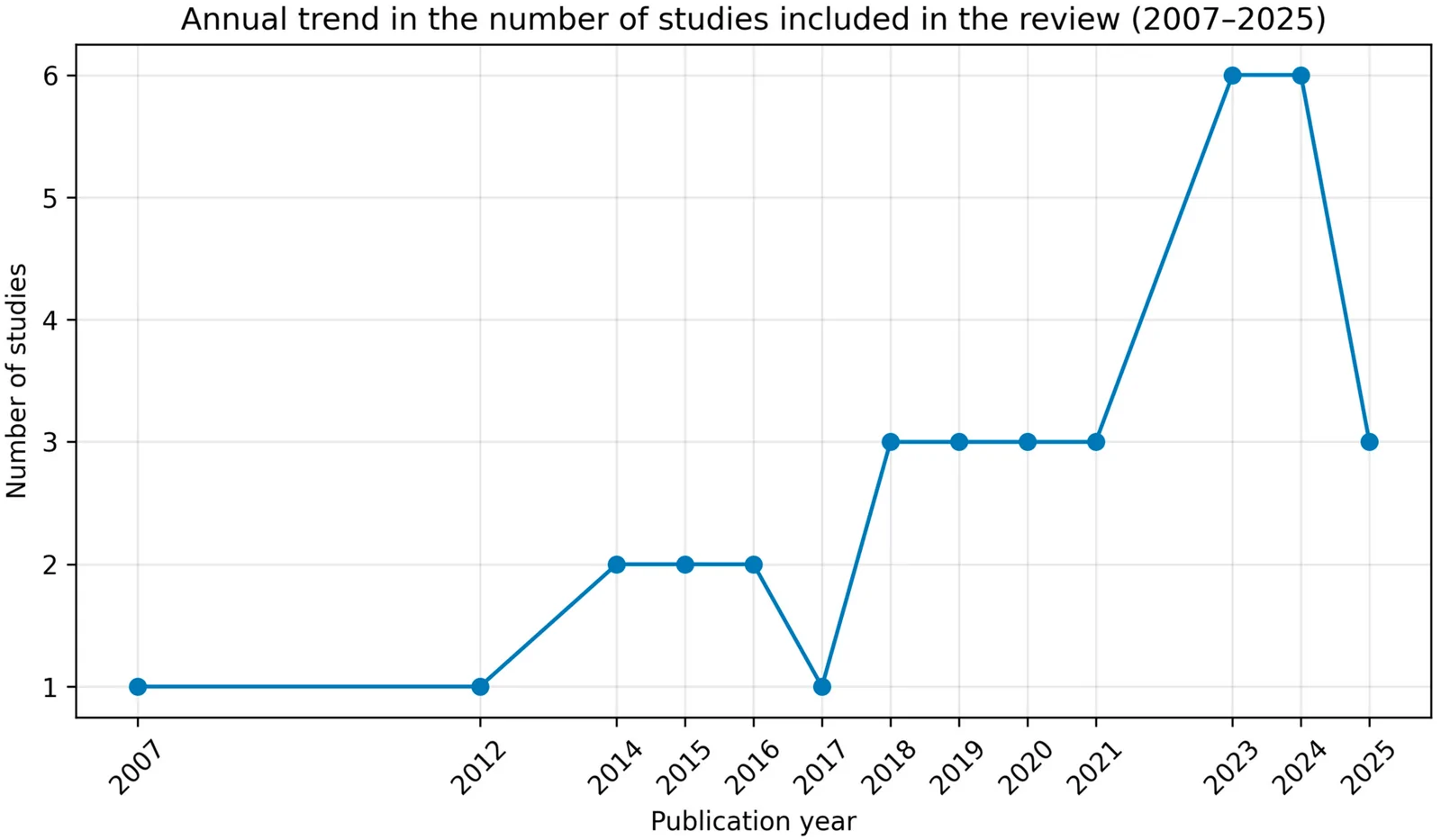
Annual trend in the number of studies included in the review (2007–2025).

The earliest study was published in 2007. There were no studies recorded from the dataset for 2008–2011, 2013, 2022, or 2026. The peak year for publications is 2024, with six studies. The second-highest year is 2023, with six studies. Figure 2 shows the annual trend in the number of studies reviewed. 

Study designs included: 18 studies were randomised controlled trials [25,26,27,28,29,30,31,33,34,35,36,37,38,39,40,41,42,43,44,45,46,47,48,49,50,51,57], three were non-randomised controlled trials [23,52,54], two were quasi-experimental [24,55], two were observational and comparative studies [27,53], one was a cross-sectional study [32], and there were two others—time series, case study, AB design [6,56].

### 3.2. Non-Invasive Behavioural Strategies Evaluated for Neurobiological Impacts

As shown in Table 1, 18 distinct non-invasive behavioural management techniques were identified. The most used methods were distraction-based techniques (strategies for diverting children’s attention from anxiety-inducing stimuli). This included the use of conventional distraction and audiovisual distractions such as VR headsets, 3D video glasses, eyeglasses, or screens [24,25,26,27,28,29,31,32,33,34,38,40,42,44,48,49,51,52,54]. Distraction was often used in combination with other techniques. The next popular strategy used was the tell–show–do technique [23,24,25,30,31,32,36,37,38,39,42,43,44,48,49,50,54], often used either alone or alongside other interventions.

Other identified techniques include modelling techniques that employed filmed, video, and in vivo modelling [30,39,43,56]; non-dental conversations [24,25,41]; dental humour [25]; playing games [37]; listening to songs [45,46,57]; and restraint [25,55]. Techniques targeting sensory regulation were 360-degree eyeglasses and VR tools [31,33,34,40,55], aimed at creating calming environments and reducing children’s resistance and fear during procedures.

One study evaluated Jacobson’s Progressive Muscle Relaxation (JPMR) and breathing control—alongside cartoon-assisted distraction—were assessed as calming strategies [53], and a few others used reinforcement-based strategies [41,42,54,56] and provided children with choices [56]. Parental involvement strategies, such as parental training and presence during treatment, were assessed in a few studies for their behavioural management impact [56]. Animal-assisted therapy (AAT) using a therapy dog [6,35,54], voice control [25,55], aromatherapy [45,47,57], threats [55] and desensitisation [56], and the Buzzy device (a vibration-based counter-stimulation device) [46] were also evaluated for their neurobiological effects.

### 3.3. Neurobiological Outcomes Assessed

The neurobiological outcomes assessed across studies are summarised in Table 2. Anxiety was the most common outcome assessed in 36 studies, followed by pain [25,29,34,45,46,48,49,52], fear [6,30,31,36,42,51,57], and stress [53]. Common biomarkers for measuring the outcomes were salivary cortisol [24,25,38], salivary alpha-amylase [55], heart rate, pulse rate [23,26,28,29,30,31,32,33,34,35,36,38,39,40,42,43,44,45,46,47,48,49,50,51,52,53,57], blood pressure [31,38,41,45,47,50,57], oxygen saturation [23,26,28,29,32,38,41,46,47,57], and respiratory rate [23]. Facial image [39] and behaviours [30,43] were also assessed.

### 3.4. Stress Biomarkers: HPA Axis Activation

Table 1 shows that salivary cortisol was the most assessed biomarker for measuring HPA axis activation. Interventions like VR distraction and music therapy [24,25,40] were associated with significant reductions in or stabilisation of cortisol levels. Recreational pre-procedural engagement prevented cortisol increases, especially in younger children [25]. In contrast, in the absence of specific BMTs, cortisol levels often spiked during dental visits, reflecting anxiety [24,25,40]. Salivary alpha-amylase (sAA), which indicates sympathetic nervous system activation, was increased during dental visits, but associations with specific BMTs were inconsistent [55]. Preliminary findings on the use of oxytocin suggest an increase associated with reduced fear [6].

### 3.5. Autonomic Nervous System Activity

As shown in Table 1, heart rate (HR) [23,26,28,29,30,31,32,33,34,35,36,39,40,41,42,43,44,45,48,49,50,51,52,53,54,56,57] and pulse rate [27,37,38,46,47,50] were used as indicators of autonomic activation; VR distraction and JPMR demonstrated positive effects in stabilising HR [32,34,40,42,53]. Other studies confirmed HR reduction following audio-visual distraction [26,28,30,38,42,44].

Blood pressure (BP) was also measured with interventions like aromatherapy [45,57], JPMR [53], tell–show–do [31,38,50], distraction [31], and music therapy [38,42,45]. Pulse rate [27,37,38,46,47,50] and oxygen saturation [23,27,29,32,38,45,46,47,50,57] were assessed in several studies, and generally remained stable—suggesting it may be less sensitive to psychological stress during treatment. Respiratory rate showed some promise as a stress indicator [23].

Pain perception was lower in children receiving BMTs, particularly with the use of VR distraction, aromatherapy, and audiovisual interventions [34,35,40,44,45,46,47,48,49,57] during procedures like local anaesthesia [45].

### 3.6. Evolution of Behavioural Interventions for Paediatric Dental Anxiety

As seen in Table 1, the studies spanned a publication timeframe from 2007 to 2025. Earlier studies (from 2014 and 2017) focused on basic behavioural management techniques such as audio-visual distraction, using cartoons or videos [26,27,28,31,33,34,38,40,42,46,48,49,50,55], tell–show–do and its modifications (tell–play-doh and ask–tell–ask) [23,24,25,32,36,37,39,42,43,44,48,49,50,55], and positive reinforcement [41,42,55,56]. Methodologically, these studies often involved smaller sample sizes ranging from 30 to 60 participants [25,27,28,31,32,34,36,37,40,41,42,43,45,47,48,51,52,54]. They relied on a limited set of biomarkers, mainly HR [28,29,30,31,32,33,34,35,36,40,42,43,44,45,46,47,48,49,50,51,52,53], BP [31,41,45,47,50], and the use of behavioural assessment tools like Frankl’s behaviour rating scale [25,32,45,46] and the Venham anxiety scale [25,27,34,35,49]. The studies employed RCTs [25,34,35,36,37,38,39,40,41,42,43,44,45,46,47,48,49,50,51,52,53,54,55,56,57], quasi-experimental [55] or non-randomised designs [52,54].

More recent contributions (2023–2025) demonstrated advances in methodology with the integration of technology. Eight studies employed VR or immersive technologies to reduce anxiety and physiological stress [32,33,34,40]. AAT emerged as a promising pilot intervention for anxiety reduction [35,54]. JPMR, An advanced relaxation approach was explored [53], while multisensory techniques combining aromatherapy with music were tested for their calming effects [43,47,57]. In addition, larger RCTs were conducted [50], using standardised cortisol and salivary alpha-amylase measurements at multiple time points (before, during, and after procedures) [25], thereby enhancing the reliability and comparability of findings across studies.

### 3.7. Behavioural and Self-Reported Measures of Anxiety and Fear

Table 2 shows the behavioural tools used to assess behavioural outcomes. These tools can be classified into four categories.

1. **Child-Reported Anxiety and Fear Instruments:** The tools comprised validated pictorial and verbal scales designed to capture the child’s subjective internal state, circumventing the limitations of literacy and developmental comprehension. *The Facial Image Scale* was the single most frequently employed instrument [23,30,31,32,33,37,38,39,41,44,45]. The *Venham picture test* and its variants—the Venham 6-point index and Venham’s rating of clinical anxiety—constituted the second most prevalent subjective measure [25,27,34,35,38,43,49,55]. *Anxiety-specific multi-item scales* were employed to provide greater granularity. The modified Child Dental Anxiety Scale (MCDAS) and its faces version, (MCDAS(f), were utilised [24,26,50]. Broader fear assessment was captured through the Dental sub-scale of the Children’s Fear Survey Schedule [28] and Corah’s Dental Anxiety Scale [54]. Regionally adapted instruments included the modified Abeer Dental Anxiety Scale—Arabic version [40], the Romanian IDAF-4C+ [53]. Notably, Kilic et al. [50] introduced the novel Child Drawing: Hospital (CD: H) scale, a projective measure that circumvents verbal response biases.
2. **Pain Perception Instruments:** Pain assessment was predominantly executed through self-report scales, with the *Wong–Baker FACES pain rating scale* featuring most prominently [24,34,46,47,49]. The *visual analogue scale* was also employed [29,51], while the *Faces pain scale—revised* was used for its improved psychometric properties over earlier iterations [42,51]. One study utilised the standard Pain Faces scale [48].
3. **Clinician/Observer-Rated Behavioural and Anxiety Scales:** Observer-rated instruments served as the bridge between subjective experience and objective clinical presentation, mitigating the limitations of child self-report in high-stress environments. *The Frankl behaviour rating scale*, particularly Wright’s modification, was the most frequently employed observer-rated tool [23,26,37,43,44]. *The FLACC scale* (*Face*, *Legs*, *Activity*, *Cry*, *Consolability*) and its revised version (r-FLACC) provided a standardised behavioural pain assessment for younger or non-verbal children [34,37,42,45,48,51]. *The Venham Anxiety and behavioural rating scales*—including the standard Venham scale, the modified Venham’s Clinical Ratings of Anxiety and Cooperative Behavior (MVARS), and the Venham 6-point index—were employed in some studies [27,40,41,46,56]. These scales offer the advantage of simultaneously rating both anxiety and cooperation. *The Houpt Scale* was selected for its comprehensive assessment of overall behaviour and treatment acceptability [26,49]. The *Sounds, Eyes, and Motor Scale* was employed in one study as an objective observer-rated anxiety measure [49]. The *Raghavendra Madhuri and Sujata pictorial scale* was also utilised in one study [36], representing a culturally adapted Indian instrument. Kaur et al., 2018 [28] additionally employed a dedicated dentist-assessed clinical anxiety rating scale alongside a Cooperative behavioural rating scale.
4. **Parental and Proxy Reports:** A minority of studies employed parent and child self-report of anxiety as parallel measures, acknowledging the importance of caregiver perspective in the paediatric dental setting [6,52]. The synthesis of findings demonstrates that while self-reported anxiety improved across interventions using tools such as the FIS, Frankl scale, and Venham scales, physiological responses showed greater variability. VR distraction was assessed using multiple tool combinations, and its impact was assessed across subjective, behavioural, and neuroendocrine domains [24,25,34,40]. Disjunctions between subjective improvement and physiological measures were also observed [6,26,28,32,49].

## 4. Discussion

This scoping review explored the neurobiological effects of non-invasive BMTs used in paediatric dentistry. Findings showed that studies examined the effects of BMTs on physiological markers of stress—including cortisol, sAA, HR, BP, and respiratory rate—and autonomic balance. The techniques explored varied, with the most used being distraction and tell–show–do. The neurobiological outcomes assessed included anxiety, pain perception, fear (*n* = 6), and stress response. The physiological regulation assessed was heart rate, oxygen saturation, blood pressure, pulse rate, respiratory rate, cortisol, and oxytocin. The behavioural assessment tools most often used were the child-reported anxiety and fear instruments. Multisensory strategies, such as combining aromatherapy with music or tactile stimulation, were also linked to reduced pain perception during invasive procedures like local anaesthesia.

A key strength of this review is its focus on an important gap in paediatric dentistry—the neurobiological mechanisms underlying BMTs—aligning with contemporary trends in interdisciplinary, trauma-informed, child-centred healthcare. The rigorous application of the PRISMA-ScR framework, a comprehensive search across five major databases using an evidence-informed strategy, and the global scope of included studies enhance generalisability. Most of the studies were randomised controlled trials, supplemented by a few quasi-experimental designs.

Nevertheless, several limitations warrant consideration. Many studies featured small samples and lacked blinding or control groups, weakening the evidence base. The restriction to English-language publications, necessitated by resource constraints and the primary authors’ language proficiency, may introduce language bias. Furthermore, the underrepresentation of studies from North America, Europe, or sub-Saharan Africa limits the overview of cultural influences on intervention acceptability. Heterogeneity in measurement tools, timing of assessments, exposure durations, and follow-up intervals further constrained comparability. A risk-of-bias assessment of the methodological quality, limitations, and strength of the evidence across the included studies would have been of value. As this was a scoping review, such objective assessment is outside the scope of the study and not considered a study limitation [58,59]. Despite these limitations, this review provides a foundation for advancing evidence-based paediatric dental care, highlighting the promise of non-invasive behavioural strategies and setting the stage for future research with more standardised, longitudinal, and culturally inclusive approaches.

Distraction techniques featured prominently across the reviewed literature, with interventions such as watching cartoons, listening to music, engaging with humour therapy, and utilising immersive tools like VR becoming increasingly popular. These techniques operate by redirecting attention away from aversive stimuli and occupying cognitive resources that would otherwise be engaged in processing fear or discomfort [60]. VR shows substantial promise by modulating physiological indicators such as HR and cortisol levels [39,61], suggesting its influence on neurobiological mechanisms related to stress regulation and the potential alteration of neural pathways involved in fear processing [62,63]. Among the technologies evaluated, the use of VR distraction was associated with significant reductions in salivary cortisol [24,25,40] and reductions in pain perception and anxiety [24,26,27,28,29,33,38,41,48,51]. The immersive nature of VR may provide a more potent distractible effect than passive screen-based viewing, potentially engaging attentional resources more fully and thereby limiting the processing of nociceptive and anxiety-provoking stimuli. These suggest a complex correlation between dental fear and anxiety symptoms, and the factors that influence dental fear [63]. More rigorous research on non-pharmacological interventions, including meta-analyses reporting on the neurobiological impact of VR distraction, is needed [62].

The tell–show–do method was the next most popularly studied BMT. It remains a cornerstone of paediatric behavioural management [64,65]. As a multimodal intervention integrating cognitive, behavioural, and exposure-based strategies, TSD draws on established learning theories, including observational learning [66]; operant conditioning, which underpins the positive reinforcement techniques frequently employed in paediatric dentistry [67]; and systematic desensitisation [68] to reduce anxiety and foster cooperation. The sequence operates by cognitively priming the child through verbal explanation (tell), reducing ambiguity; allowing vicarious exposure that challenges catastrophic beliefs via demonstration (show); and engaging the child in the actual procedure using reinforcement to build self-efficacy (do). This predictability has been hypothesised to promote emotional regulation and reduce stress responses, as suggested by lower cortisol levels and heart rate [23,28,50]. TSD outperforms live modelling alone in lowering fear and pulse rate [30] and enhances pain tolerance when combined with distraction tools such as VR [31,34]. Through gradual exposure, TSD mirrors systematic desensitisation principles, introducing stimuli in stages to minimise fear, as demonstrated by lowered autonomic arousal [50]. Direct evidence of neural activity, such as changes in amygdala or prefrontal cortex function, was, however, not measured in the included studies.

TSD’s effectiveness may vary with age, working best for children aged three and older [69], and across cultural contexts, where parental presence may either ease or heighten anxiety [12,70]. Its success depends on the clinician’s communication skills and emotional attunement [71], as well as the use of developmentally appropriate interventions [67], making TSD as much a relational tool as a procedural one. Its simplicity and adaptability across developmental stages, ability to modulate neurobiological stress pathways, and potential synergy with technologies such as VR position it as a powerful tool for trauma-informed, child-centred dental care.

Modelling strategies—whether through live demonstration, video, or peer observation—continue to gain prominence as low-cost, developmentally appropriate interventions that transform dental care experiences through social learning [52], with the potential to reduce anticipatory fear and enhance child cooperation through vicarious learning [72]. This may result in lower activation of stress pathways and decreased cortisol release [13,17]. Bandura’s key processes—attention, retention, reproduction, and motivation—are directly engaged [66]: animated cartoons draw the child’s focus; observed behaviours are encoded and recalled during their appointment; children imitate these behaviours and are motivated to do so when rewarded or praised. The effectiveness of modelling is supported both by the current review and other systematic reviews [29,56], indicating that it can prevent, reduce, and reverse fear responses [61]. Physiological data further reinforce modelling’s neurobiological impact, demonstrating reduced salivary cortisol levels and stabilised HR and BP, indicating reduced sympathetic nervous system arousal [73]. The diurnal cortisol patterns and their relevance to dental stress response measurement have implications for standardising timing in biomarker collection [73]. Modelling can also effectively manage behaviour in children with special needs, such as autism, with an indirect positive impact on parents [74]. Cultural tailoring of content further enhances its effectiveness and use in diverse populations [70].

However, modelling tends to be most effective in children with mild to moderate anxiety; those with severe dental phobia may require adjunctive interventions [41]. Parental modelling may backfire if the parent displays visible dental fear, inadvertently transferring anxiety to the child [25]. Research gaps persist, particularly regarding neurobiological underpinnings. Few studies have used neuroimaging to examine mirror neuron involvement during observational learning in dental contexts, with one study showing that at the neural level, observational learning is mediated by the mirror neuron system, a network of cortical neurons that fire both when an individual performs an action and when they observe another individual performing the same action. This vicarious neural simulation allows the child to pre-experience the dental procedure in a safe, controlled manner, effectively priming the brain’s somatosensory and motor representations of the dental encounter without exposing the child to actual threat [75].

However, several research gaps remain. Few studies have directly employed neuroimaging to examine mirror neuron system engagement during modelling interventions in the dental setting. The potential role of oxytocin in modulating the effectiveness of modelling, given oxytocin’s known role in enhancing social salience and trust, remains largely unexplored [76]. In addition, the differential effectiveness of live versus filmed modelling, and the optimal duration and frequency of modelling exposure, require further investigation. Future research directions may include hybrid approaches combining modelling with VR-based immersive experiences, culturally tailored modelling content for diverse populations [77,78], and standardised protocols for measuring mirror neuron system engagement using EEG or functional neuroimaging. Addressing these gaps would clarify the neurobiological pathways through which modelling exerts its anxiolytic effects and facilitates the development of evidence-based, neurobiologically informed behaviour guidance protocols.

A notable finding across studies was that reductions in cortisol, HR, and BP coincided with improved anxiety and fear scores, supporting the validity of multi-modal assessment to evaluate intervention effects. However, variability in measurement tools, timing, and biomarker collection protocols was evident. Studies highlighted the importance of procedure timing, such as biomarker levels spiking at specific phases (e.g., injection) [79], indicating the need for standardised timing in future research. The effectiveness of behaviour management varied by technique: VR and BWE had strong effects on autonomic markers, while modelling and TSD primarily influenced behavioural outcomes, sometimes with accompanying physiological changes. Relaxation techniques primarily targeted physiological regulation.

A consistent finding across multiple studies was the divergence between self-reported or observer-rated anxiety—which almost uniformly improved with behavioural interventions—and physiological measures, which showed variable, often non-significant, or even paradoxical responses. This discrepancy was particularly evident for heart rate and pulse rate, where improvements in subjective anxiety were not always accompanied by corresponding reductions in autonomic arousal. This phenomenon may reflect the complex and sometimes decoupled nature of subjective anxiety and its physiological correlates, particularly in paediatric populations where developmental factors may influence the relationship between cognitive appraisal and autonomic responding.

Vincent et al.’s [6] finding that alpha-amylase followed the trend of oxytocin more than cortisol is noteworthy. Alpha-amylase is a surrogate marker of sympathetic–adrenal–medullary axis activity [80], whereas cortisol reflects HPA axis activity [81]. The observation that alpha-amylase tracked with oxytocin—a hormone associated with social bonding and stress buffering—rather than with cortisol, suggests that certain interventions, particularly those involving social or animal interaction, may modulate stress through pathways not simply reducible to HPA axis suppression. This raises the possibility that different behavioural interventions engage distinct neurobiological circuits: technology-based distractions may predominantly influence HPA axis activity (as reflected by cortisol reductions), while socially enriched interventions may engage oxytocinergic and sympathetic–adrenal–medullary axis pathways in more complex ways. This postulation needs further investigation.

Also, a review of the compiled assessment toolkit reveals several salient methodological features. The high reliance on the Facial Imaging Scale and the Venham scales underscores the field’s preference for rapid, child-friendly, pictorial formats that minimise fatigue and maximise compliance in young, potentially distressed populations. A clear two-tiered approach to anxiety assessment emerges: broad screening instruments (CFSS-DS, MCDAS) are complemented by procedure-specific state anxiety measures (FIS, VPT), enabling both trait and state characterisation. The combination of the FLACC (pain behaviour) and Frankl (cooperation) scales provides comprehensive observational coverage of the two principal dimensions of procedural distress. A distinct subset of higher-fidelity studies integrates objective physiological monitoring with neuroendocrine biomarkers, moving beyond peripheral autonomic measures (heart rate, blood pressure) into the central neuroendocrine domain (cortisol, alpha-amylase, oxytocin) [6,24,25,40]. This triangulation permits a more nuanced understanding of the differential engagement of stress-regulatory systems. Future studies on the neurobiological effects of BMT may want to explore this approach. More recent studies are, however, exploring the use of novel approaches integrating technology, reflecting a shift toward interactive, child-friendly interventions. AAT has emerged as a promising pilot intervention for anxiety reduction [82], while multisensory techniques are also being tested [83]. Telehealth adaptations have facilitated parent-led anxiety interventions, especially for neurodiverse populations such as children with autism [84]. There has also been a shift toward low-contact strategies. Future studies could explore the use of the CARD™ system [32] to reduce pain during childhood vaccination [78,85].

Furthermore, the substantial heterogeneity in study designs, outcome measures, and intervention protocols limits the ability to draw definitive conclusions. As noted in a recent systematic review of technology-enhanced behaviour guidance, very high heterogeneity, frequent risk of bias, limited safety reporting, and sensitivity to small-study effects indicate that the magnitude of benefit is uncertain and likely context-dependent [86]. The included studies vary widely in sample sizes, age ranges, dental procedures, control conditions, and outcome assessment time points. Many studies are underpowered to detect differences in physiological parameters, and few report effect sizes or confidence intervals.

These gaps warrant attention. First, standardisation of physiological outcome measures and reporting would facilitate meta-analytic synthesis. Second, longitudinal studies are needed to determine whether the neurobiological effects observed during a single visit translate into sustained reductions in dental anxiety and improved oral health behaviours over time. Third, comparative effectiveness research directly comparing different intervention modalities within the same study population would help clarify which strategies are most effective for which patient subgroups. Fourth, the neurobiological mechanisms underlying these interventions remain poorly understood; studies that incorporate multiple biomarkers (cortisol, alpha-amylase, oxytocin, heart rate variability) within a single protocol would help elucidate the differential engagement of stress-regulatory systems.

## 5. Conclusions

This scoping review demonstrates that non-invasive BMT in paediatric dentistry may be associated with beneficial effects on subjective anxiety, pain perception, and behavioural cooperation, with variable effects on objective neurobiological and physiological measures. Virtual reality distraction emerges as the most consistently effective technology-based intervention. The discrepant findings between subjective and physiological measures highlight the complexity of the stress response in paediatric dental settings and underscore the need for multi-method assessment approaches. The current evidence base is limited by heterogeneity and methodological concerns. The overall pattern of findings suggests BMT can reduce the neurobiological and psychological burden of dental care for children. Further studies are needed to identify how to integrate BMT into routine paediatric dental practice for these purposes.

## Figures and Tables

**Table 1:** Characteristics of the included studies.

S/N	Author(s) and Year of Publication	Country	Study Design	Sample Size	Age (Years)	Behavioural Management Interventions	Neurological Outcome	Biological Outcome
1.	Vincent et al., 2020 [6]	USA	Time-series design	18	8–12 years	Animal-assisted therapy (dog)	Fear and anxiety	Cortisol level, oxytocin level, salivary alpha-amylase
2.	Raseena et al., 2020 [23]	India	Non-randomised controlled trial	90	6–9 years	Tell–show–do	Anxiety	Heart rate oxygen saturation respiratory rate
3.	Shetty et al., 2019 [24]	India	Quasi-experimental study	120	5–8 years	Non-medical conversation, tell–show–do, conventional distraction, voice control	Anxiety	Salivary cortisol level
4.	Suresh & Shetty, 2024 [25]	India	Randomised controlled trial	40	8–15 years	Non-dental conversation, dental humour, tell–show–do, mild restraints and routine distractions	Pain, anxiety	Salivary cortisol level
5.	Nuvvula et al., 2015 [26]	India	Randomised controlled trial	90	7–10 years	Audiovisual distraction	Anxiety	Heart rate
6.	Prabhakar et al., 2007 [27]	India	Comparative study	60	4–8 years	Audio distraction and audio-visual distraction	Anxiety	Pulse rate and oxygen saturation
7.	Kaur et al., 2018 [28]	India	Randomised controlled trial	60	4–8 years	Audiovisual distraction	Anxiety	Heart rate
8.	Agarwal et al., 2017 [29]	India	Randomised controlled trial	120	3–14 years	Audiovisual distraction	Pain and anxiety	Heart rate and oxygen saturation
9.	Vishwakarma et al., 2021 [30]	India	Randomised controlled trial	98	5–7 years	Live modelling, and tell–play–do	Fear and anxiety	Heart rate
10.	Pande et al., 2020 [31]	India	Randomised controlled trial	60	5–8 years	Tell–show–do, audiovisual distraction (VR and mobile phone game distraction)	Fear and anxiety	Blood pressure and heart rate
11.	Babu et al., 2024 [32]	India	Cross-sectional study	40	6–10 years	Comfort, Ask, Relax, and Distract (CARD™) System and tell–show–do	Anxiety	Oxygen saturation and heart rate
12.	Varshitha et al., 2023 [33]	India	Randomised controlled trial	93	6–11 years	Distraction with VR Eyewear	Anxiety	Heart rate
13.	Pathak et al., 2023 [34]	India	Randomised controlled trial	30	6–12 years	Distraction with VR device	Anxiety and pain	Heart rate
14.	Thakkar et al., 2021 [35]	India	Randomised controlled trial	102	5–10 years	Animal-assisted therapy	Anxiety	Heart rate
15.	Elicherlia et al., 2024 [36]	India	Randomised controlled trial	50	7–11 years	Tell–show–do, ask–tell–do	Anxiety and fear	Heart rate
16.	Radhakrishna et al., 2019 [37]	India	Randomised controlled trial	60	4–8 years	Smartphone dentist game, tell–show–do	Anxiety	Pulse rate
17.	Khandelwal et al., 2018 [38]	India	Randomised controlled trial	400	5–8 years	Tell–show–do, audio-visual distraction	Anxiety	Blood pressure, pulse rate oxygen saturation
18.	Karekar et al., 2019 [39]	India	Randomised controlled trial	63	7–9 years	Tell–show–do and live and filmed modelling	Anxiety	Facial image Heart rate
19.	Bagher et al., 2023 [40]	Saudi Arabia	Randomised controlled trial	36	6–14 years	Distraction with VR	Anxiety	Salivary cortisol level and heart rate
20.	Al-khotani et al., 2016 [41]	Saudi arabia	Randomised controlled trial	56	7–9 years	Verbal communication and positive reinforcement	Anxiety	Blood pressure and heart rate
21.	Mitrakul et al., 2015 [42]	Thailand	Randomised controlled trial	48	4–16 years	Distraction with audio-visual (AV eyeglasses), tell–show–do, positive reinforcement, and conventional distraction	Fear and anxiety	Blood pressure and Heart rate
22.	Paryab & Arab 2014 [43]	Iran	Randomised controlled trial	46	4–6 years	Tell–show–do, filmed modelling	Anxiety	Heart rate Behaviour
23.	Abbasi et al., 2021 [44]	Pakistan	Clinical trial	160	6–11 years	Distraction with mobile application “little lovely dentist”, YouTube dental video songs, tell–show–do	Anxiety	Heart rate
24.	Abdalhai et al., 2024 [45]	Syria	Randomised controlled trial	56	6–10 years	Aromatherapy with music	Anxiety and pain	Blood pressure, heart rate, and oxygen saturation
25.	Mohammed & Omer, 2023 [46]	Egypt	Randomised controlled trial	90	5–11 years	Buzzy device and upbeat music	Anxiety and pain	Pulse rate, oxygen saturation
26.	Omer et al., 2024 [47]	Egypt	Randomised controlled trials	45	4–7 years	Aromatherapy	Anxiety	Pulse rate, oxygen saturation
27.	El-Sharkawi et al., 2012 [48]	Egypt	Randomised controlled trial	48	5–7 years	Tell–show–do, distraction with audiovisual video glasses	Pain and anxiety	Heart rate
28.	Üstün et al., 2025 [49]	Turkey	Randomised controlled trial	62	4–9 years	Cartoon-assisted visual/auditory distraction, tell–show–do	Anxiety and pain	Heart rate
29.	Kilic et al., 2025 [50]	Turkey	Randomised controlled trial	150	6–12 years	Tell–show–do	Anxiety	Blood pressure, pulse rate, oxygen saturation
30.	Bagattoni et al., 2018 [51]	Italy	Randomised controlled trial	48		Audio-visual distraction using video eyeglasses	Fear and anxiety	Heart rate
31.	Guinot et al., 2014 [52]	Spain	Non-randomised controlled trial	34	6–8 years	Distraction with audio-visual technique (cartoon film)	Pain and anxiety	Heart rate
32.	Bucur et al., 2025 [53]	Switzerland	Observational study	189	8–17 years	Jacobson’s progressive muscle relaxation and breathing techniques	Anxiety and stress	Heart rate, blood pressure
33.	Pinheiro et al., 2023 [54]	Brazil	Non-randomised controlled trial	59	4–14 years	Animal-assisted therapy (dog)	Anxiety	Heart rate
34.	Reis et al., 2016 [55]	Brazil	Quasi-experimental	69	4–12 years	Tell–show–do positive reinforcement, distraction, modelling, and aversive (voice control, physical containment, threat for responsible leave, request for responsible leave) techniques	Anxiety	Salivary alpha-amylase
35.	Vernice, 2024 [56]	New York	Single case	1	6 years	Parent training intervention, positive reinforcement, desensitisation, incorporating special interest, in vivo modelling, and providing choice.	Anxiety	Heart rate
36.	Janthasila & Keeratisiroj, 2023 [57]	Thailand	Randomised controlled trial	128	10–12 years	Aromatherapy, music therapy	Fear, anxiety	Heart rate, blood pressure, oxygen saturation

**Table 2:** Summary of studies on the neurobiological effects of non-invasive behavioural management strategies in paediatric dentistry.

S/N	Author(s)	Tools to Measure Study Outcomes	Findings
1	Vincent et al., 2020 [6]		Oxytocin trended positively from baseline for most participants, though not statistically significantly. Cortisol decreased over the three time points, while alpha-amylase followed the trend of oxytocin more than cortisol.
2	Raseena et al., 2020 [23]	Wright’s modification of Frankl’s behaviour rating scale and Facial Image Scale	Tell–show–do, when used with virtual tools, showed lower anxiety and improved cooperation during dental procedures than when tell–show–do was used alone.
3	Shetty et al., 2019 [24]	Revised modified child cental anxiety scale, Wong–Baker FACES pain rating scale	Reduction in pain perception, state anxiety and salivary cortisol in children using VR distraction.
4	Suresh & Shetty, 2024 [25]	Venham’s picture test and Frankl’s behaviour rating scale	Report on pain between groups, dental anxiety, and behaviour between dental visits was better in children for whom VR distraction was used compared to those using conventional behaviour management. Salivary cortisol levels were lower at pre-treatment for children for whom VR distraction was used, and lower post-treatment for children for whom VR was used, though both groups had lower cortisol levels post treatment.
5	Nuvvula et al., 2015 [26]	MCDAS(f), Wright’s modification of Frankl behaviour rating scale and Houpt scale	MCDAS(f) and the Houpt scale showed significant reduction in the group that used behaviour guidance techniques and audiovisual compared to those using behaviour guidance techniques and audio only and those using only basic behaviour guidance techniques. Pulse rate increased significantly in the three groups.
6	Prabhakar et al., 2007 [27]	Venham’s picture test, Venham’s rating of clinical anxiety	Audiovisual distraction technique was more effective in managing anxious paediatric dental patients compared to the audio distraction technique.
7	Kaur et al., 2018 [28]	Dental sub-scale of children’s fear survey schedule-short scale; dentists’ assessment of Clinical anxiety rating scale and co-operative behavioural rating scale.	No significant difference in pulse rate of the control group, audio-only group, and audiovisual group before, during, and after the procedures at first, second, and third visits. The audiovisual group showed significantly lower scores than the audio-only group and the control group. The audio-only group also showed a significantly lower score than the control group.
8	Agarwal et al., 2017 [29]	Visual analogue scale	Eutectic mixture of local anaesthetic cream and benzocaine (20%) gel was least effective in reducing the pain during the needle insertion when used without audiovisual aids compared to when used with audiovisual aids.
9	Vishwakarma et al., 2021 [30]	Facial Image Scale, Venham-6-point index	Pulse rate, Facial Image Scale, and Venham-6-point index scores were significantly lower among children who received tell–play–do intervention when compared to those who received live modelling intervention.
10	Pande et al., 2020 [31]	Frankl’s rating scale, Facial Image Scale	Physiological and non-physiological parameters significantly decreased post-intervention in all the groups—tell–show–do, audio distraction, audiovisual distraction and mobile phone game distraction—with a maximum decrease in the groups that received audiovisual distraction.
11	Babu et al., 2024 [32]	Facial Image Scale	No statistically significant differences were observed in the physiological parameters (oxygen saturation levels and pulse rates), emotional responses, and behavioural responses pre- and post-procedure.
12	Varshitha et al., 2023 [33]	Facial Image Scale	Postoperative pulse rate and Facial Image Scale score in the VR + modified tell–show–do group reduced significantly, while they increased significantly in the modified tell–show–do only group.
13	Pathak et al., 2023 [34]	Venham’s picture test, Wong–Baker FACES pain rating scale, and FLACC scale	At baseline, Venham’s picture test and heart rate showed no statistically significant difference in the groups using and not using a VR device. There was a statistically significant increase in post-procedure heart rates in the group not using a VR device, with no significant change in the group using a VR device.
14	Thakkar et al., 2021 [35]	Venham picture test	Pulse rate and the Venham picture test score were significantly lower in children who interacted with therapy dogs than in those who had dental treatment carried out in a regular dental setup.
15	Elicherla et al., 2024 [36]	Raghavendra Madhuri and Sujata pictorial scale	Children in the tell–show–do group had significantly lower heart rates and RMS-PS scores in intra-group comparisons. However, children in the ask–tell–ask group showed a significant reduction only in the RMS-PS scores but not in heart rate.
16	Radhakrishna et al., 2019 [37]	Face, leg activity, cry, consolability (FLACC) rating scale, Frankl’s behavioural scale, Facial Imaging Scale	The pulse rates, Facial Imaging Scale and FLACC scores were lower in more children with a Frankl’s behaviour rating score of 4, and there was better operator compliance in both the Tell–Show–Play-doh and smartphone dentist game groups than in the conventional tell–show–do group.
17	Khandelwal et al., 2018 [38]	Facial Image Scale, Venham’s picture test	Audiovisual distraction reduced anxiety better than tell–show–do. Combining tell–show–do and audiovisual distraction had an additive effect in the reduction in anxiety level.
18	Karekar et al., 2019 [39]	-Facial Image Scale	Children who underwent tell–show–do, live and filmed modelling during diagnosis and preventive dental care had significantly lower anxiety scores and heart rates after diagnosis and preventive dental care, except for the heart rate during diagnosis.
17	Bagher et al., 2023 [40]	Venham anxiety and behavioural rating scale, modified version of the Abeer Dental Anxiety Scale–Arabic version	At the end of the treatment, the salivary cortisol level was significantly lower in the VR distraction group when compared with those watching a video cartoon on a regular screen. Neither the Venham anxiety and behavioural rating scale nor the heart rate significantly differed between the groups.
18	Al-Khotani et al., 2016 [41]	Facial Image Scale, modified Venham’s clinical ratings of anxiety and cooperative behavior scale (MVARS)	The audiovisual distraction group showed significantly lower MVARS scores than the control group during treatment. The pulse rate was significantly increased in the control group during injection with local anaesthesia, but not in the audiovisual distraction group.
19	Mitrakul et al., 2015 [42]	Faces pain scale—revised, face, legs, activity, crying and consolability scale (FLACC)	The use of audiovisual eyeglasses reduced the heart rate and the FLACC score during pre-operation and the first use of a high-speed handpiece. Heart rate decreased during rubber dam placement, the first use of a high-speed handpiece, and during remaining treatment in the second visit as compared with the first visit with or without wearing the eyeglasses.
20	Paryab and Arab, 2014 [43]	Venham and Frankl’s rating scale	There were no statistically significant differences in heart rate measures, clinical anxiety, and cooperative behaviour scores between those who received tell–show–do at the first visit and treatment procedures performed by the dentist for the children at second visit vs. children who watched a film consisting of the procedure of tell–show–do performed on a child model at the first visit and treatment procedures performed by the dentist for the children at second visit.
21	Abbasi et al., 2021 [44]	Facial Image Scale, pulse oximeter, Frankl’s behavioural rating scale	Marked reduction in heart rate and Facial Image Scale scores were found in patients who received mobile applications and dental video songs post-operatively. An increase in heart rate and Facial Image Scale scores was seen in the tell–show–do and the control group post-operatively.
22	Abdalhai et al., 2024 [45]	Facial Image Scale, Facial Leg Activity Cry Consolability (FLACC) scale.	Dental anxiety and heart rate, and diastolic and systolic blood pressure were significantly lower in the aromatherapy with music group when compared to the control group, with no differences in pain perception and oxygen saturation between groups.
23	Mohammed & Omer, 2023 [46]	Venham clinical anxiety rating scale and Wong-Becker Faces pain rating scale.	Music significantly reduced pain and anxiety before, during, and after anaesthesia, while the Buzzy device had a more pronounced effect during the procedure. Both interventions led to significant changes in pulse rate, but oxygen saturation remained unaffected.
24	Omer et al., 2024 [47]	Wong–Baker scale	There was a statistically significant reduction in the mean scores of the Wong–Baker scale, pulse rate, oxygen saturation, and blood pressure in children with no interference before dental procedures, children who inhaled three drops of rosemary oil for 3 min, and children who inhaled two drops of lemongrass oil for 3 min before, during, and after dental procedures.
25	El-Sharkawi et al., 2012 [48]	Pain Faces scale, the Face, legs, activity, cry, consolability (FLACC)	The pain scores in the Pain Faces scale and the FLACC scale were significantly lower when the use of audiovisual distraction was used compared to when it was not used.
26	Üstün et al., 2025 [49]	Venham picture test, Wong–Baker FACES pain rating scale, Sounds, Eyes, and Motor Scale, and Houpt Scale.	The cartoon-assisted distraction technique did not significantly reduce anxiety compared to the tell–show–do method. A non-significant reduction in pain perception was observed during local anaesthesia with distraction. However, this technique significantly reduced self-reported pain during treatment and improved child cooperation and behaviour.
27	Kilic et al., 2025 [50]	Modified Child Dental Anxiety Scale Faces Version (MCDASf), Child Drawing: Hospital	Compared to the coloured coat group, MCDAS_f_ values recorded before both appointments were higher in the white coat group. Also, the CD: H values of the coloured coat group were significantly lower after preventive dental treatments. There were significantly lower pulse rates after, rather than before, appointments in both coat groups. The oxygen saturation was lower in the coloured coat group after dental examination and after preventive dental treatment. Diastolic blood pressure was lower after dental examination compared to before dental examination in the coloured coat group.
28	Bagattoni et al., 2018 [51]	Face pain scale—revised (FPS-R), the revised Face, Leg, Activity, Cry, and Consolability scale (r-FLACC), visual analogue scale	The mean FPS-R score and the mean r-FLACC score were significantly lower using only the audio–video distraction during the second clinical session. The use of video eyeglasses also significantly reduced the operator’s stress measured with the visual analogue scale.
29	Guinot et al., 2014 [52]	Parent and child self-report of anxiety	Significant improvement in the behaviour of children shown cartoon films. Also, there was a significant increase in heart rate when the anaesthetic was injected. No statistically significant differences existed between the visits in terms of parental perception of the patient’s anxiety, or the patient’s self-reported anxiety, pain and heart rate.
30	Bucur et al., 2025 [53]	The Romanian version of the IDAF-4C+	Jacobson’s Progressive Muscle Relaxation led to the highest reductions in IDAF-4C^+^ scores and systolic blood pressure compared to breathing control. Breathing control showed moderate anxiety reduction with minor physiological changes. High anxiety–strong responders mainly benefited from JPMR; while those with moderate anxiety were partial responders and those with low anxiety were non-responders.
31	Pinheiro et al., 2023 [54]	Corah’s Dental Anxiety Scale	Significant reduction in heart rate in the AAT group compared to children who were conditioned by methods routinely used in the clinic. The heart rate of children conditioned by methods routinely used in the clinic did not change before, during, or after treatment. Children conditioned by methods routinely used in the clinic showed a significant increase in the Corah Dental Anxiety Score before and after treatment. In the AAT group, there was no change in Corah Dental Anxiety scores before and after treatment.
32	Reis et al., 2016 [55]	Venham picture test	Salivary alpha-amylase showed high and moderate levels before the dental procedure in most children. Behavioural management techniques were not associated with Venham picture test scores and salivary alpha-amylase activity.
33	Vernice, 2024 [56]	Venham anxiety and behaviour rating scale	The child’s Venham anxiety and behaviour rating scale score decreased, and cooperation during the dental exam increased
34	Janthasila & Keeratisiroj, 2023 [57]	None	The control group had increased heart rate. The music therapy group had decreased dental anxiety and fear as well as systolic blood pressure. The aromatherapy experimental group had increased oxygen saturation. The group receiving music therapy combined with aromatherapy had decreased dental anxiety and fear, heart rate, and systolic and diastolic blood pressure, with increased oxygen saturation values. Music therapy combined with aromatherapy had a co-influence on dental anxiety, fear, and oxygen saturation.

## Data Availability

No new data were created or analysed in this study. Data sharing is not applicable to this article.

## Supplementary Materials

The following supporting information can be downloaded at: https://www.mdpi.com/article/10.3390/clinpract16090160/s1, Table S1: PRISMA-ScR Checklist; Table S2: Search Strategies used in the databases.

## Abbreviations

The following abbreviations are used in this manuscript:

BMT	Behaviour Management Technique
PRISMA	Preferred Reporting Items for Systematic Reviews and Meta-Analyses
ANS	Autonomic Nervous System
HPA	Hypothalamic–Pituitary–Adrenal
RCTs	Randomised Controlled Trials
OSF	Open Science Framework
